# Characteristics, prognosis, and treatment response in HFpEF patients with high vs. normal ejection fraction

**DOI:** 10.3389/fcvm.2022.944441

**Published:** 2022-09-09

**Authors:** Jun Gu, Jia-han Ke, Yue Wang, Chang-qian Wang, Jun-feng Zhang

**Affiliations:** Department of Cardiology, Shanghai Ninth People's Hospital, Shanghai Jiaotong University School of Medicine, Shanghai, China

**Keywords:** heart failure with preserved ejection fraction, left ventricular ejection fraction, prognosis, treatment response, phenogroups

## Abstract

**Background:**

Heart failure with preserved ejection fraction (HFpEF) patients varied by left ventricular ejection fraction (LVEF) have different clinical characteristics, prognosis, and treatment response. With data from our prospective HFpEF cohort, we assessed the possible relationship between clinical characteristics, outcome as well as treatment response and LVEF.

**Methods:**

We compared differences in baseline characteristics and clinical outcomes across LVEF categories (50%≤LVEF <60% vs. LVEF≥60%) in 1,502 HFpEF patients, and determined whether LVEF modified the treatment response. During 5-year follow-up, all-cause mortality was used as the primary endpoints, and composite endpoints (all-cause mortality or HF hospitalization) were set as the secondary endpoint.

**Results:**

Patients with higher LVEF were statistically older, more likely to be women and have a history of atrial fibrillation. Patients with lower LVEF category were more likely to have a history of coronary artery disease. The incidences of all-cause mortality and composite endpoints were higher in patients with higher LVEF. Also, LVEF modified the spironolactone treatment effect for the primary outcome and secondary endpoint with stronger estimated benefits at the lower LVEF category with respect to all-cause mortality (HR 0.734, 95% CI 0.541–0.997, *P* = 0.048) and all-cause mortality or HF hospitalization (HR 0.767, 95% CI 0.604–0.972, *P* = 0.029).

**Conclusion:**

The characteristics and outcomes of HFpEF patients varied substantially by LVEF. Patients with higher LVEF encountered more adverse events than those with lower LVEF. The potential efficacy of spironolactone was greatest at the lower category of LVEF spectrum in HFpEF.

## Introductions

Heart Failure with preserved ejection fraction (HFpEF) accounts for the majority of heart failure (HF) patients in the elderly. Our understanding of HFpEF was in a giant leap in recent decades while some controversies still exist in its treatments (1). HFpEF is characterized by the coexistence of a series of systemic metabolic or inflammatory disorders that contribute to coronary endothelial dysfunction, microvascular rarefaction and cardiac fibrosis, which eventually result in an impaired left ventricular distensibility (2). The role of renin-angiotensin-aldosterone system (RAAS) inhibitors in HFpEF remains to be determined since multiple clinical studies showed conflicting results (1, 2). The PARAGON-HF trial of angiotensin receptor-neprilysin inhibitor (ARNI) did not present a significantly lower rate of hospitalization for HF and death from cardiovascular causes in patients with left ventricular ejection fraction (LVEF) ≥ 45%, but it suggested possible benefits among female patients as well as those with an LVEF ≤ 57% (3). In the EMPEROR-PRESERVED trial, prescription of sodium-glucose cotransporter 2 inhibitor (SGLT2i) empagliflozin demonstrated a reduction in risk of composite cardiovascular death or total HF hospitalization in HF with LVEF > 40% (4). The benefit was driven by a reduction in HF hospitalizations, but it cannot be applied to patients with an LVEF of more than 60% in subgroup analysis (3). Therefore, HFpEF patients, as classified by LVEF, might have different clinical characteristics, prognosis and treatment response.

Given survival detriments observed in individuals with supra-normal LVEF, a disease phenotype termed heart failure with supra-normal ejection fraction (HFsnEF) has been defined in patients with LVEF > 65% (5–7). Unfortunately, there is still no established treatment strategy for HFpEF when LVEF lies on the higher category (≥60%), even though the number of such patients is expected to increase globally as society ages. This is an issue that needs to be further investigated and resolved.

In the present study, we utilized data from our prospective HFpEF cohort to assess the relationship between clinical characteristics, outcome as well as treatment response and LVEF.

## Methods

### Study design and patient enrollment

HFpEF patients derived from our prospective HFpEF cohort study that has previously been described (8–10). HFpEF was defined by clinical features of HF with LVEF ≥50% (1). LVEF was determined using biplane modified Simpson's measurements by echocardiography. Recruitment occurred when the patient was in the hospital for a primary diagnosis of HFpEF (the assessment was performed following stabilization of the acute HF) or in the outpatient setting with an episode of decompensated HF (requiring hospitalization or treatment in an outpatient setting) within 3 months. Patients were excluded when they met one or more of the exclusion criteria which contained severe valve disease, transient acute pulmonary edema in the context of primary acute coronary syndrome, end-stage renal failure [estimated glomerular filtration rate (eGFR) < 30 mL/min/1.73 m^2^], specific HF subgroups (including constrictive pericarditis, congenital heart disease, hypertrophic cardiomyopathy, cardiac amyloid, and chemotherapy-associated cardiomyopathy), isolated right HF, and life-threatening co-morbidity with life expectancy <1 year. Besides all of these, patients were not considered eligible when their initial LVEF is <50% but thereafter improved to the designated level (≥50%) during the index admission. All participants were informed of the purpose of the study and provided written informed consent. Investigations were in strictly accordance to the Declaration of Helsinki and were approved by the institutional ethics committee.

### Endpoints

The primary outcome was defined as all-cause mortality. The secondary outcome was composite endpoints of death or HF hospitalization.

### Follow-up

Enrolled patients were followed for 5 years. The majority of patients visited our out-patient clinic at a frequency of at least every 3 months, and they were interviewed annually by telephone when they were absent of the scheduled visit. We prospectively collected information on deaths, hospitalizations for HF. Death was classified as cardiovascular, non-cardiovascular or unknown reasons, and death or HF hospitalization was adjudicated by an independent blinded physician. When primary or secondary endpoint occurred to the specific patient, the time duration was calculated from the initial date of the start of follow-up.

### Statistical analysis

Statistical analysis was performed using SPSS Statistical Software, Version 22.0 (SPSS Inc., Chicago, IL, USA). Arithmetic means ± standard deviations were calculated for quantitative variables, while qualitative variables were given as frequency and percentage. *T*-test was used for quantitative variable analysis and a two-sided χ^2^ test was operated to compare qualitative variables and differences in clinical endpoints. Cox proportional hazards regression model was used to explore the association between risk factors and the risk of all-cause mortality or composite endpoints. All the predictors with a significance of *P* ≤ 0.10 in the univariable analysis and forced inclusion variables that were recognized as strong predictors of clinical endpoints were entered into a specific multivariable model. Hazard ratios (HRs) and corresponding 95% confidence intervals (CIs) were used as reported. Freedom from the occurrence of all-cause mortality or composite endpoints at 5 years was analyzed with Kaplan–Meier statistics, with differences assessed using the log-rank test. All values were two-tailed, and a *P* < 0.05 was considered statistically significant.

## Results

### Clinical characteristics stratified by LVEF

A total of 1,929 patients were potentially eligible for the study from January 2007 to December 2016, 116 were unable to provide informed consent, and a further 311 met one or more of the study exclusion criteria, leaving 1,502 patients included in the study. Enrolled patients consisted 40.5% women and 59.5% men, with a mean age of 69.8 ± 6.6 years. For the prevalence of cardiovascular diseases, 70.8% of the enrolled patients had a history of hypertension and 37.8% had atrial fibrillation (AF). Type 2 diabetic mellitus (T2DM) occurred in 37.2% of the patients and the overall mean estimated glomerular filtration rate (eGFR) was 60.6 ± 9.3 mL/min/1.73 m^2^. All clinical characteristics, as stratified by LVEF (group 1: 50 ≤ LVEF < 60%, group 2: LVEF ≥ 60%), were shown in Table 1. Group 1 was composed of younger individuals (69.5 ± 6.3 years) and a higher prevalence of coronary artery disease (CAD) (40.1%) and percutaneous coronary intervention (PCI) procedure (24.4%). While group 2 was consisted of patients with older ages (70.2 ± 6.9 years), higher proportion of women (46.6%), higher prevalence of AF (40.2%) and higher level of systolic blood pressure (133.3 ± 10.2 mmHg). Both groups had similar rates of beta-blockers, spironolactone and angiotensin converting enzyme inhibitor/angiotensin II receptor blocker (ACEI/ARB) prescription treatment assignments.

**Table 1:** Baseline characteristics.

	**LVEF (≥50% and <60%)**	**LVEF (≥60%)**	***P***
	**(Group 1, *n* = 731)**	**(Group 2, *n* = 771)**	
Age (years)	69.5 ± 6.3	70.2 ± 6.9	0.046
Women (gender)	283 (38.7)	359 (46.6)	0.002
BMI (kg/m^2^)	24.8 ± 2.2	24.7 ± 2.2	0.257
**Medical history**			
CAD	293 (40.1)	260 (33.7)	0.011
Prior PCI	178 (24.4)	14 (19.1)	0.013
Prior CABG	29 (4.0)	30 (3.9)	0.939
Hypertension	530 (72.5)	533 (69.1)	0.151
T2DM	277 (37.9)	282 (36.6)	0.598
Atrial fibrillation	258 (35.3)	310 (40.2)	0.050
Stroke	86 (11.8)	82 (10.6)	0.488
COPD	82 (11.2)	72 (9.3)	0.230
Smoking	240 (32.8)	230 (29.8)	0.210
Dyslipidemia	243 (33.2)	225 (29.2)	0.090
**Medications**			
ACEI/ARB	498 (68.1)	509 (66.0)	0.385
Beta-blocker	428 (58.5)	451 (58.5)	0.983
Spironolactone	181 (24.8)	213 (27.6)	0.207
Anticoagulant	74 (10.1)	96 (12.5)	0.155
Antiplatelet	381 (52.1)	369 (47.9)	0.099
Statin	273 (37.3)	253 (32.8)	0.066
**Clinical status**			
NYHA (I/II/III/IV)	77/249/363/42	90/277/349/55	0.326
Heart rate (bpm)	80.7 ± 8.2	79.9 ± 8.4	0.063
Systolic BP (mmHg)	131.7 ± 11.8	133.3 ± 10.2	0.006
Diastolic BP (mmHg)	79.8 ± 8.8	79.1 ± 8.7	0.088
**Laboratory variables**			
eGFR (ml/min/1.73 m^2^)	60.7 ± 9.1	60.5 ± 9.4	0.670
Hemoglobin (g/dL)	11.9 ± 1.5	11.9 ± 1.4	0.370
BNP (pg/mL)	782.1 ± 339.7	762.2 ± 291.4	0.220
**Echo data**			
LVEF (%)	55.3 ± 2.3	63.0 ± 2.9	<0.001
LAD (mm)	41.9 ± 3.8	42.3 ± 3.8	0.261
LVMI (g/m^2^)	118.4 ± 10.9	118.7 ± 11.3	0.697
E/e'	13.2 ± 1.9	13.1 ± 1.9	0.261

Data are presented as mean ± SD or number (%) of subjects.

BMI, body mass index; CAD, coronary artery heart disease; PCI, percutaneous coronary intervention; CABG, coronary artery bypass graft; T2DM, type 2 diabetes mellitus; COPD, chronic obstructive pulmonary disease; ACEI/ARB, angiotensin converting enzyme inhibitor/angiotensin II receptor blocker; NYHA, New York Heart Association functional class; BP, blood pressure; eGFR, estimated glomerular filtration rate; BNP, B-type natriuretic peptides; LVEF, left ventricular ejection fraction; LAD, left atrium diameter; LVMI, left ventricular mass index; E/e', mitral Doppler early velocity/mitral annular early velocity.

### Prognostic relationship between LVEF and clinical outcome

**Figure 1:**
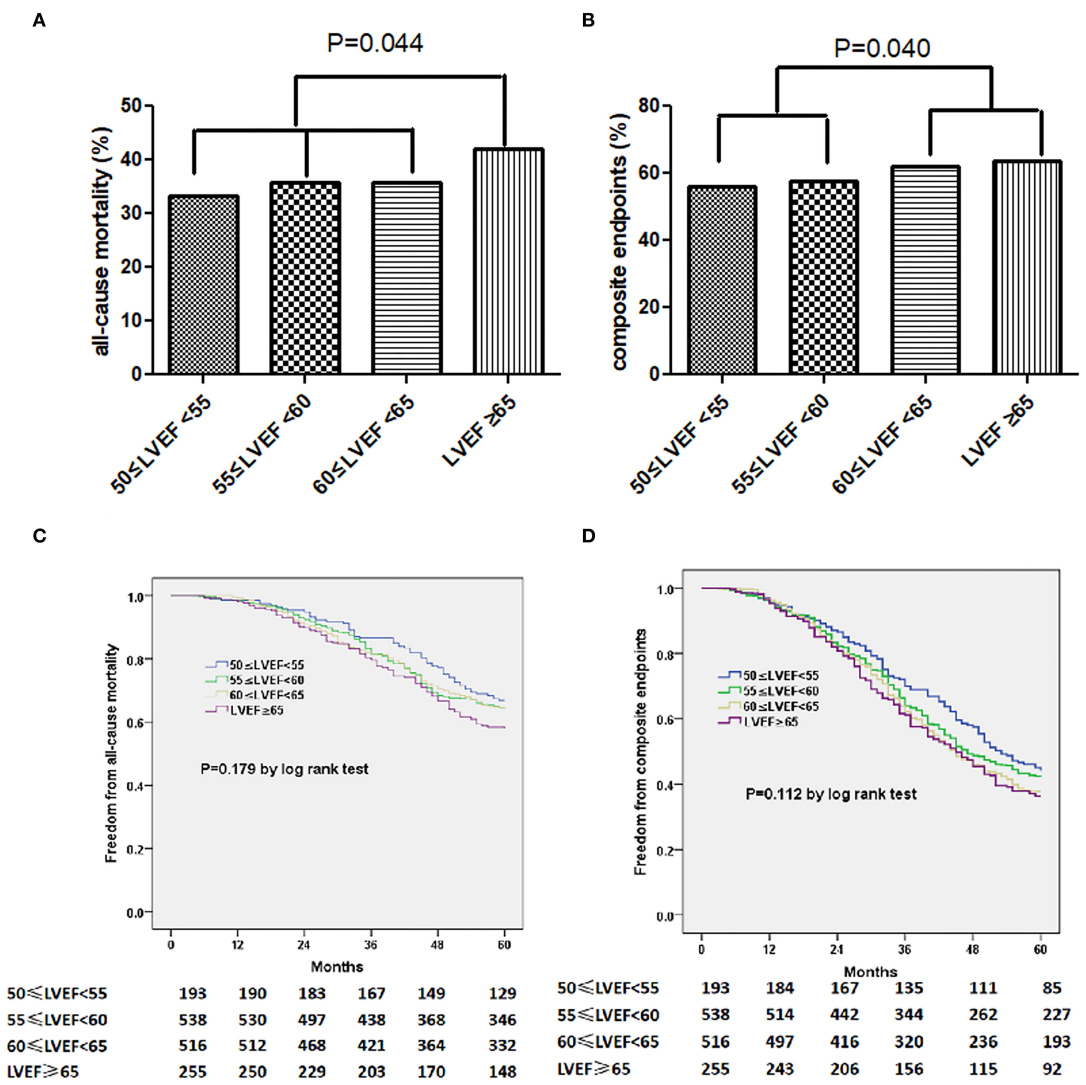
The incidence of all-cause mortality **(A)** for composite endpoints **(B)** stratified by LVEF. Kaplan-Meier curves of freedom from all-cause mortality **(C)** or composite endpoints **(D)** varied by LVEF. The numbers at the bottom of the figure are “number at risk.”

The primary endpoints of all-cause death occurred in 547 (36.4%) patients, and the secondary endpoints of all-cause mortality or HF hospitalization were observed in 901 of the 1,502 (60.0%) participant. We additionally observed a distinct pattern of association between LVEF and risk of composite endpoints on 5-year follow-up (group 1 vs. group 2: *P* = 0.040). Patients with LVEF ≥ 65% had a higher cumulative incidence of all-cause mortality comparing those with a LVEF < 65% (*P* = 0.044, Figure 1A). This pattern of association was also similar in subgroup from LVEF < 60% to LVEF ≥ 60% on the decreased incidence of all-cause mortality or HF hospitalization (*P* = 0.040, Figure 1B). And a higher incidence of mortality (HR: 1.378, 95% CI 1.011–1.878, *P* = 0.043) and composite endpoints (HR: 1.284, 95% CI 1.006–1.638, *P* = 0.044) was documented in patients with LVEF of ≥65% comparing those with LVEF of 50–55%. Unadjusted Kaplan–Meier estimators illustrated the stratification of survival by LVEF (Figures 1C,D). Using multivariable adjusted Cox models, we reported that LVEF ≥ 60% was an independent risk factor for composite endpoints (HR 1.149, 95% CI 1.006–1.313, *P* = 0.040, Table 3). Besides, older age or higher E/e' level was sufficient for independently predicting occurrence of all-cause mortality, so does the prediction of composite endpoints using ages (Tables 2, 3).

**Table 2:** Multivariable cox analysis for all-cause mortality in total HFpEF patients.

	**HR**	**95%CI**	***P***
Age	1.017	1.004–1.030	0.010
Female	0.870	0.732–1.034	0.115
BMI	1.023	0.985–1.063	0.242
CAD	1.072	0.900–1.277	0.434
T2DM	0.875	0.733–1.044	0.139
Hypertension	1.040	0.863–1.253	0.683
AF	1.092	0.918–1.298	0.319
BNP category	1.095	0.987–1.215	0.087
Hemoglobin	1.034	0.975–1.097	0.264
eGFR	1.000	0.991–1.009	0.992
NYHA class	0.952	0.855–1.061	0.375
Spironolactone	0.850	0.698–1.036	0.108
ACEI/ARB	0.874	0.733–1.042	0.133
Betablocker	0.966	0.815–1.146	0.694
E/e'	1.051	1.006–1.098	0.027
LVEF (<60 vs. ≥60%)	1.118	0.943–1.325	0.199

HFpEF, heart failure with preserved ejection fraction; BMI, body mass index; CAD, coronary artery disease; T2DM, type 2 diabetes mellitus; AF, atrial fibrillation; BNP, B-type natriuretic peptides; eGFR, estimated glomerular filtration rate; NYHA, New York Heart Association functional class; ACEI/ARB, angiotensin converting enzyme inhibitor/angiotensin II receptor blocker; E/e', mitral Doppler early velocity/mitral annular early velocity; LVEF, left ventricular ejection fraction.

**Table 3:** Multivariable Cox for composite endpoints in total HFpEF patients.

	**HR**	**95%CI**	***P***
Age	1.018	1.007–1.028	0.001
Female	0.990	0.866–1.132	0.884
BMI	1.022	0.992–1.052	0.159
CAD	1.112	0.971–1.274	0.126
T2DM	1.135	0.992–1.298	0.066
Hypertension	0.982	0.851–1.134	0.810
AF	1.098	0.960–1.257	0.172
BNP category	1.063	0.980–1.152	0.141
Hemoglobin	0.984	0.940–1.029	0.477
eGFR	0.994	0.987–1.001	0.105
NYHA class	0.973	0.893–1.060	0.532
Spironolactone	0.883	0.758–1.028	0.109
ACEI/ARB	0.894	0.778–1.026	0.111
Betablocker	0.907	0.795–1.035	0.149
E/e'	1.025	0.990–1.061	0.166
LVEF (<60 vs. ≥60%)	1.149	1.006–1.313	0.040

HFpEF, heart failure with preserved ejection fraction; BMI, body mass index; CAD, coronary artery disease; T2DM, type 2 diabetes mellitus; AF, atrial fibrillation; BNP, B-type natriuretic peptides; eGFR, estimated glomerular filtration rate; NYHA, New York Heart Association functional class; ACEI/ARB, angiotensin converting enzyme inhibitor/angiotensin II receptor blocker; E/e', mitral Doppler early velocity/mitral annular early velocity; LVEF, left ventricular ejection fraction.

Of 547 deaths during the study, 344 (62.9%) were ascribed to cardiovascular, 177 (32.4%) to non-cardiovascular, and 26 (4.8%) to unknown causes. Of cardiovascular deaths, 115 (33.4%) were due to sudden death, 127 (36.9%) to HF, 39 (11.3%) to stroke, 26 (7.6%) to myocardial infarction, and 37 (10.8%) to other cardiovascular causes. Rates of cardiovascular (*P* < 0.001) and sudden death (*P* = 0.010) were higher in those with lower LVEF (<60%), while rates of non-cardiovascular death (*P* < 0.001) were greater in patients with higher LVEF (≥60%).

AF and HFpEF share common pathophysiologic features, both syndromes share overlapping symptoms. About 37.8% of enrolled HFpEF patients had a history of AF, after ruling out AF, we found that patients with elevated LVEF tended to have a trend of increased composite endpoints (*P* = 0.072). However, there was no significant difference in terms of all-cause mortality.

### Effects of medical therapy on mortality or composite endpoints stratified by LVEF

**Figure 2:**
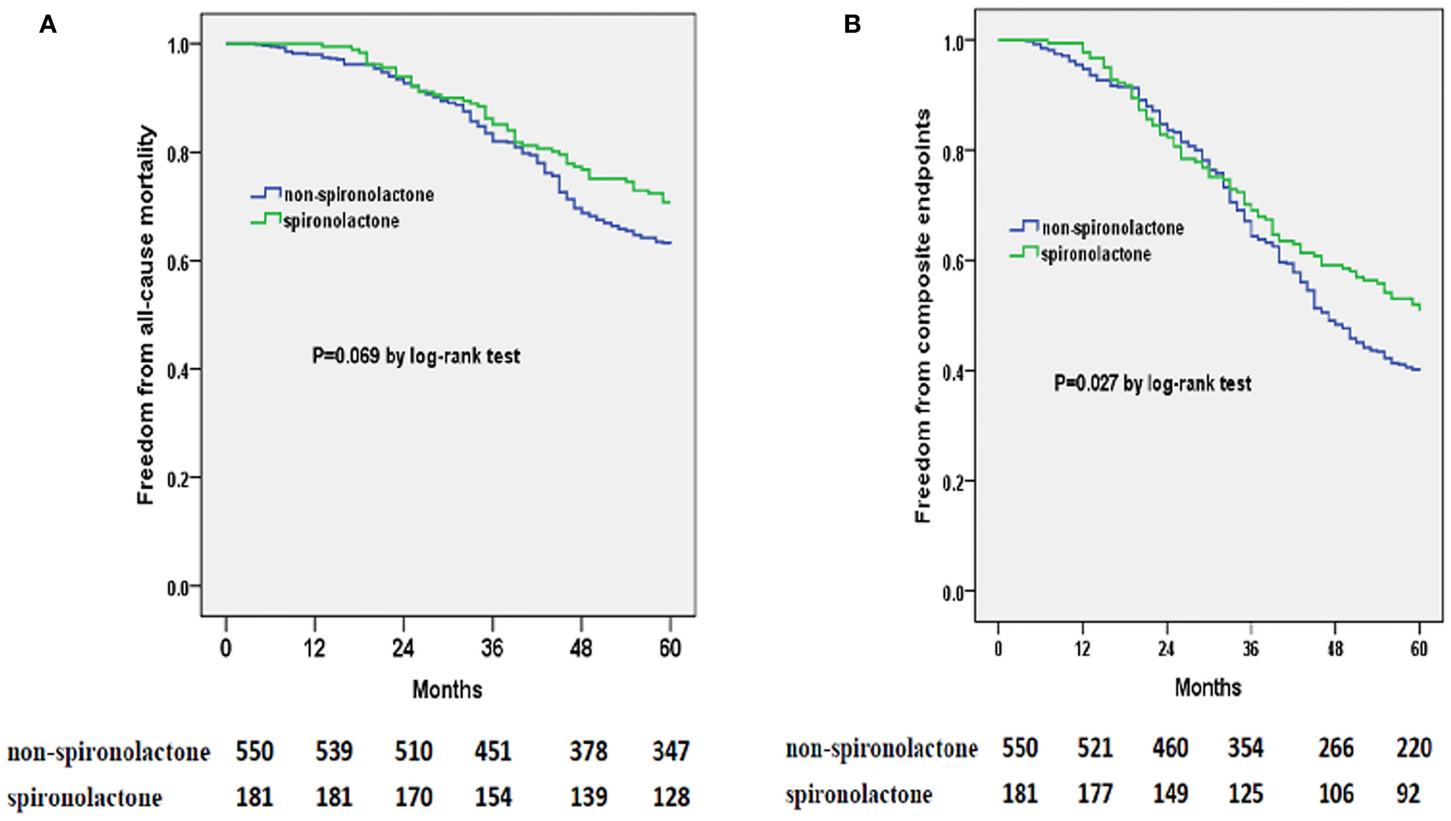
Kaplan-Meier curves of freedom from all-cause mortality **(A)** and composite endpoints **(B)** for spironolactone or non-spironolactone group in HFpEF patients with LVEF < 60% during 5 years of following-up. The numbers at the bottom of the figure are “number at risk.”

Totally, none of ACEI/ARB, beta-blockers or spironolactone therapy was evidenced efficient in lowering risks of 5-year all-cause mortality or composite endpoints in the whole cohort study (Tables 2, 3). However, from results of the stratified analyses, we found that spironolactone was relevant with a significantly lower risk of all-cause mortality and composite endpoints in the group of LVEF <60% instead of LVEF ≥ 60% (Tables 4–7), but beta-blockers or ACEI/ARB did not appear to substantially benefit the both subgroups (Tables 4–7). Moreover, spironolactone prescription was also accompanied with a reduced risk for composite endpoints by log-rank test in patients with LVEF < 60% (Figure 2).

**Table 4:** Multivariable cox analysis for all-cause mortality in HFpEF patients with LVEF <60%.

	**HR**	**95%CI**	***P***
Age	1.014	0.994–1.034	0.183
Female	0.795	0.613–1.032	0.085
BMI	1.017	0.961–1.077	0.553
CAD	1.189	0.927–1.524	0.172
DM	0.802	0.618–1.040	0.096
Hypertension	1.154	0.866–1.538	0.329
AF	1.183	0.913–1.532	0.204
BN pCategory	1.199	1.027–1.400	0.021
Hemoglobin	1.027	0.944–1.118	0.532
eGFR	0.994	0.980–1.008	0.373
NYHA class	0.983	0.834–1.158	0.836
Spironolactone	0.734	0.541–0.997	0.048
ACEI/ARB	0.807	0.622–1.047	0.106
Beta-blocker	0.820	0.639–1.053	0.120
E/e'	1.088	1.017–1.263	0.014
LVEF (<55 vs. ≥55%)	1.125	0.841–1.504	0.429

HFpEF, heart failure with preserved ejection fraction; BMI, body mass index; CAD, coronary artery disease; T2DM, type 2 diabetes mellitus; AF, atrial fibrillation; BNP, B-type natriuretic peptides; eGFR, estimated glomerular filtration rate; NYHA, New York Heart Association functional class; ACEI/ARB, angiotensin converting enzyme inhibitor/angiotensin II receptor blocker; E/e', mitral Doppler early velocity/mitral annular early velocity; LVEF, left ventricular ejection fraction.

**Table 5:** Multivariable Cox analysis for composite endpoints in HFpEF patients with LVEF <60%.

	**HR**	**95%CI**	***P***
Age	1.021	1.005–1.037	0.010
Female	1.015	0.832–1.239	0.881
BMI	1.018	0.973–1.064	0.442
CAD	1.150	0.946–1.399	0.162
T2DM	1.195	0.981–1.455	0.077
Hypertension	1.032	0.829–1.284	0.781
AF	1.102	0.900–1.350	0.347
BNP category	1.051	0.931–1.185	0.421
Hemoglobin	0.972	0.909–1.039	0.400
eGFR	0.994	0.984–1.005	0.315
NYHA class	0.928	0.815–1.056	0.256
Spironolactone	0.767	0.604–0.972	0.029
ACEI/ARB	0.834	0.679–1.024	0.082
Beta-blocker	0.844	0.695–1.026	0.089
E/e'	1.051	0.996–1.108	0.068
LVEF (<55 vs. ≥55%)	1.082	0.864–1.355	0.494

HFpEF, heart failure with preserved ejection fraction; BMI, body mass index; CAD, coronary artery disease; T2DM, type 2 diabetes mellitus; AF, atrial fibrillation; BNP, B-type natriuretic peptides; eGFR, estimated glomerular filtration rate; NYHA, New York Heart Association functional class; ACEI/ARB, angiotensin converting enzyme inhibitor/angiotensin II receptor blocker; E/e', mitral Doppler early velocity/mitral annular early velocity; LVEF, left ventricular ejection fraction.

**Table 6:** Multivariable Cox analysis for all-cause mortality in HFpEF patients with LVEF ≥60%.

	**HR**	**95%CI**	***P***
Age	1.020	1.002–1.037	0.028
Female	0.932	0.737–1.178	0.556
BMI	1.031	0.978–1.087	0.260
CAD	0.990	0.771–1.273	0.939
T2DM	0.920	0.722–1.173	0.502
Hypertension	0.954	0.744–1.223	0.710
AF	1.017	0.803–1.287	0.890
BNP category	1.003	0.868–1.159	0.968
eGFR	1.003	0.990–1.016	0.659
Hemoglobin	1.030	0.946–1.120	0.499
NYHA class	0.917	0.790–1.066	0.259
Spironolactone	0.977	0.749–1.275	0.867
ACEI/ARB	0.886	0.695–1.129	0.329
Beta-blocker	1.102	0.869–1.398	0.422
E/e'	1.032	0.973–1.095	0.293
LVEF (<65 vs. ≥65%)	1.241	0.971–1.587	0.085

HFpEF, heart failure with preserved ejection fraction; BMI, body mass index; CAD, coronary artery disease; T2DM, type 2 diabetes mellitus; AF, atrial fibrillation; BNP, B-type natriuretic peptides; eGFR, estimated glomerular filtration rate; NYHA, New York Heart Association functional class; ACEI/ARB, angiotensin converting enzyme inhibitor/angiotensin II receptor blocker; E/e', mitral Doppler early velocity/mitral annular early velocity; LVEF, left ventricular ejection fraction.

**Table 7:** Multivariable cox analysis for composite endpoints in HFpEF patients with LVEF ≥60%.

	**HR**	**95%CI**	***P***
Age	1.015	1.001–1.028	0.031
Female	0.969	0.808–1.162	0.734
BMI	1.029	0.989–1.072	0.161
CAD	1.078	0.890–1.305	0.444
T2DM	1.064	0.883–1.283	0.513
Hypertension	0.932	0.768–1.131	0.475
AF	1.079	0.898–1.296	0.417
BNP category	1.088	0.972–1.217	0.143
Hemoglobin	0.989	0.927–1.055	0.729
eGFR	0.993	0.983–1.003	0.149
NYHA class	1.027	0.912–1.157	0.658
Spironolactone	1.004	0.818–1.232	0.969
ACEI/ARB	0.937	0.774–1.134	0.501
Beta-blocker	0.931	0.775–1.119	0.449
E/e'	1.008	0.963–1.056	0.722
LVEF (<65 vs. ≥65%)	1.093	0.900–1.328	0.370

HFpEF, heart failure with preserved ejection fraction; BMI, body mass index; CAD, coronary artery disease; T2DM, type 2 diabetes mellitus; AF, atrial fibrillation; BNP, B-type natriuretic peptides; eGFR, estimated glomerular filtration rate; NYHA, New York Heart Association functional class; ACEI/ARB, angiotensin converting enzyme inhibitor/angiotensin II receptor blocker; E/e', mitral Doppler early velocity/mitral annular early velocity; LVEF, left ventricular ejection fraction.

## Discussion

In patients with HFpEF enrolled in this prospective cohort study, we observed marked differences in baseline characteristics based on LVEF within the preserved range. The primary endpoint of all-cause mortality as well as the composite endpoints of all-cause mortality or HF hospitalization were more statistically frequent in subgroup of patients with a higher end of the LVEF spectrum. The potential benefit of spironolactone with respect to adverse events was greatest in patients with LVEF < 60%.

Previous study has indicated a u-shaped relationship between Hazard ratios (HRs) for adverse cardiovascular events and LVEF, in which a nadir falls in the range of 60–65% and all the other intervals had significantly higher HRs (5). Besides, a higher mortality in both inpatients and outpatients with HF and even those without a diagnosis of HF has been predicted by LVEF ≥ 70%, even after adjusting varies confounders (5). After a median follow-up time of 5.6 years, women with supra-normal (≥65%) LVEF indicated a higher likelihood of developing major adverse cardiovascular events (MACEs) than women with normal (55–65%) LVEF (11). Considering the significant sex- and age-specific differences in baseline LVEF with an overall higher LVEF and stronger age-dependent increments in LVEF observed in women, an association between increased mortality and supra-normal LVEF has been implicated in the female population (12, 13). Indeed, recent data indicated that women with CAD and supra-normal LVEF are more likely to experience heightened risk of both short-term and long-term mortality (14, 15). Patients with supra-normal LVEF encountered more MACEs than those with normal LVEF (16). Our results also demonstrated that, along with the increased LVEF in HFpEF patients, a similar sex- and age-specific differences and higher incidence of primary or secondary outcome were observed. The mode of death differs LVEF in the present study was consistent with previous study. Previous studies also showed that cardiovascular death, particularly sudden death accounts for a greater proportion in those with LVEF below the range of normal (17, 18). Unlike our results, rates of all-cause death were higher in those with lower LVEF (18), which might be related to the different cut-off points of LVEF grouping.

HFpEF, as is acknowledged clinically, represents a heterogeneous group of disease processes. This heterogeneity may underly difficulties identifying effective treatments for HFpEF. Our previous study described three HFpEF phenogroups based on model-based clustering (8). Phenogroup 1 consists of younger individuals, in which the classification of New York Heart Association class (NYHA), renal function, left ventricular mass index (LVMI) and cardiac diastolic function are relatively preserved, with a low the prevalence of type 2 diabetes mellitus (T2DM) and CAD and a high level of hemoglobin. While the patients in Phenogroup 2 are comparatively older, characterized concomitantly by higher proportion of women and higher incidence of AF. Middle aged patients are more likely to be in Phenogroup 3, and their body mass index (BMI) are usually higher, so does the prevalence of CAD and T2DM and the severity of HF symptoms assessed by NYHA. The cumulative incidence of all-cause mortality or composite endpoints was highest in phenogroup 3 followed by phenogroup 2 and phenogroup 1. Paralleled to our previous study, the prevalence of CAD was higher in the group of patients with a LVEF between 50 and 60%, while the prevalence of AF was higher in group of patients with LVEF ≥ 60%, and these patients were relatively older. Additionally, clinical endpoints were more likely to occurred in patients with LVEF ≥ 60%.

As for the treatment response, our previous trials demonstrated that patients instructed with beta-blockers were significantly less likely to develop both all-cause mortality and composite endpoints, and ACEI/ARB therapy provides patients with a markedly lower risk of composite endpoints in HFpEF phenogroup 3 which documented with higher incidence of CAD and T2DM, and that might be accounted for the favorable effects of beta-blockers and ACEI/ARB (8). In the present study, we found that the effect of spironolactone on the primary outcome or composite endpoints varied by baseline LVEF such that the greatest potential benefit was observed in patients with LVEF <60%. From that perspective, spironolactone was suggested for therapies on HF with reduced ejection fraction (HFrEF) due to its improved outcomes (1). However, data regarding the effects of spironolactone on HFpEF was inconclusive and evidence for reductions in mortality is lacking (19–23). The TOPCAT trial failed to provide the conclusion of an overall benefit in the primary composite outcome of cardiovascular death or HF hospitalization among HFpEF patients with spironolactone therapy. However, in an exploratory analysis operated in patients solely in the United States, a small benefit on the primary outcome was noticed, indicating that spironolactone was associated with a reduced risk of HF hospitalization in TOPCAT and TOPCAT-Americas subgroup (19). An analysis of the TOPCAT trial using machine learning identified a phenotype that was characterized by obesity, diabetes, renal disease and inflammation, which exhibited a higher incidence of cardiovascular events and a better treatment-response to spironolactone (20). For those HFpEF patients involved in TOPCAT trial (LVEF ≥ 45%), their baseline characteristics and outcomes also varied substantially by LVEF. And the potential efficacy of spironolactone was most significant in those with a low LVEF (23). Differed from the randomized controlled trial of TOPCAT, our observational study tended to be a real-world study. Given the LVEF of more than 50% in enrolled patients, our results better reflect the efficacy of spironolactone for HFpEF in clinical practice. We may also speculate that this particular HFpEF subgroup, as is more likely to be associated with structural heart disease and volume overload, may be more sensitive to spironolactone treatment.

Moreover, one of our previous retrospective study concluded that the consistent exposure of spironolactone in hypertensive patients is strongly associated with benefits of lower incidence of left ventricular hypertrophy, left ventricular diastolic dysfunction and the new-onset HFpEF (24). Besides, other studies provided that in specific phenogroup with high burden of comorbidities and severe HF symptoms, a lower incidence of the primary composite outcome and HF hospitalization can be expected with spironolactone prescription (20, 25).

Further precise analyses stratified by LVEF had been carried out in EMPEROR-PRESERVED trial, which presented that empagliflozin has an additional property of reducing risks of composite cardiovascular death or total HF hospitalization in HF with LVEF > 40%. However, the benefits did not extend beyond LVEF of 60% (26). The PARAGON-HF trial, which examined the efficacy of sacubitril/valsartan in HFpEF patients, also reported that the preventive effect on HF hospitalization was weakened in patients with a higher LVEF (2).

Previous study (11) gave an underlying explanation to illustrate the increased incidence of mortality in patients with supra-normal LVEF, that the reduced coronary flow reserve (CFR) and a blunted heart rate reserve (HRR) after adenosine were possibly connected with supra-normal LVEF. Also, the occurrence of microvascular dysfunction and the increase in sympathetic suggesting tone might be responsible for the worse outcomes in female patients among all participants with a high LVEF. In addition, there is another study reported that one of the mechanisms triggering reduction of CFR in patients with supra-normal LVEF may be the neurovascular hyperactivity at rest and inadequate reserve at stress, since they are manifested with decreased HRR, higher non-corrected myocardial blood flow (ncrMBF), and a subsequent reduced non-corrected (ncCFR) (16). Secondly, the upregulation on cardiomyocyte oxygen demand after the hyperdynamic workload in supra-normal LVEF patients may be underlying myocardial injury, microvascular ischemia, interstitial fibrosis, impaired cardiac mechanics and the detected reduction of CFR. Supra-normal LVEF or microvascular dysfunction might be an underlying explanation to illustrate the increased incidence of adverse events in HFpEF patients with higher LVEF, and there are other unknown mechanisms to be investigated.

## Limitations

First, the main limitation lies on the of the observational nature of the study design. The prescription of beta-blockers, ACEI/ARB or spironolactone to corresponding patients was based on the own decision of the responsible physician and the risk factors were not equally distributed among the prespecified groups. Therefore, a sufficiently powerful randomized clinical trial is needed to give a further proof. Second, the patients were enrolled from a single academic center and the number are relatively small, which might affect the generalizability of results. Third, we regretted that we did not routinely measure left atrium volume index (LAVI) during the follow-up of the study. Fourth, the treatment of HFpEF have been significantly improved during the research period, such as ARNI and SGLT2i. However, no participants in our study prescribed ARNI, and only a very small number of patients received SGLT2i prescriptions. Lastly, the variability of LVEF determination could not be entirely averted. Our echocardiography tests were performed at a single echocardiography laboratory, which had followed strict standards of practice such that an LVEF assessment likely had high internal validity. According to our internal statistics, the variation in measurements between the two investigators was 3.5% and intra-observer variability was 2.8%.

## Conclusion

In patients with HFpEF enrolled in this prospective cohort, patient characteristics and outcomes varied substantially by LVEF. Patients with LVEF ≥ 60% encountered more adverse outcomes than those with LVEF < 60%. The potential efficacy of spironolactone was greatest at the lower category of the LVEF spectrum.

## Data availability statement

The original contributions presented in the study are included in the article/supplementary material, further inquiries can be directed to the corresponding authors.

## Ethics statement

The studies involving human participants were reviewed and approved by the Ethics Committee of Shanghai Ninth People's Hospital, Shanghai Jiaotong University School of Medicine. The patients/participants provided their written informed consent to participate in this study.

## Author contributions

JG designed the research. J-hK and YW collected the data. JG and J-fZ analyzed the data. JG and C-qW wrote the manuscript. All authors contributed to the article and approved the submitted version.

## Funding

This study was supported by the National Natural Science Foundation of China (82070381), Clinical Research Program (JYLJ202014), Multidisciplinary Team (201911) and Biobank for Coronary Heart Disease (YBKA201910) of Shanghai Ninth People's Hospital, Research Projects from the Natural Science Foundation of Shanghai (20ZR1431100).

## Conflict of interest

The authors declare that the research was conducted in the absence of any commercial or financial relationships that could be construed as a potential conflict of interest.

## Publisher's note

All claims expressed in this article are solely those of the authors and do not necessarily represent those of their affiliated organizations, or those of the publisher, the editors and the reviewers. Any product that may be evaluated in this article, or claim that may be made by its manufacturer, is not guaranteed or endorsed by the publisher.
